# *Periplaneta americana* extract alleviates steatohepatitis in a mouse model by modulating HMGB1-mediated inflammatory response

**DOI:** 10.3389/fphar.2022.995523

**Published:** 2022-10-03

**Authors:** Yang Xiao, Chongqing Gao, Junru Wu, Jing Li, Lijuan Wang, Yang You, Tianqi Peng, Keke Zhang, Mingrong Cao, Jian Hong

**Affiliations:** ^1^ Department of Hepatological Surgery, The First Affiliated Hospital, Jinan University, Guangzhou, China; ^2^ Department of Pathophysiology, School of Medicine, Jinan University, Guangzhou, China

**Keywords:** steatohepatitis, alcohol consumption, obesity, periplaneta americana extractions (PAEs), high-mobility group box1 (HMGB1)

## Abstract

Alcoholic abuse and obesity are the most common lifestyle implications of chronic liver injury, and always act synergistically to increase the risk of mortality. *Periplaneta americana* has a long history of being applied in medicine, including wound healing, antitumor, antibacterial, antiviral, antifibrotic, and cardiomyocyte-protecting. Ganlong capsule (GLC), a natural prescription drug extracted from *Periplaneta americana*, has been widely used in HBV-related symptoms. However, the anti-steatohepatitis efficacy and mechanisms of GLC have not yet been characterized. Here, we found the protective effect of GLC on the development of hepatic steatosis, oxidative stress, and inflammation *in vivo* under alcohol exposure combined with a high-fat and high-cholesterol diet (HFHC). Consistently, GLC exhibited a hepatoprotective property by preventing hepatocytes from oxidative stress injury and lipid accumulation *in vitro*. In addition, it exerted an anti-inflammation characteristic by reducing macrophage recruitment and decreasing the expression of pro-inflammatory genes *in vivo* and *in vitro*. Mechanically, GLC serum, isolated from GLC-treated mice, reduced extracellular high-mobility group box 1 (HMGB1) of dying hepatocytes; and suppressed subsequent M1 polarization of macrophages in the co-culture system. Furthermore, GLC serum inhibited inflammatory response *via* suppressing the HMGB1 release and blocking the downstream TLR4/NF-kB pathway. Collectively, GLC alleviates steatohepatitis induced by alcohol consumption and obesity through inhibition of the HMGB1-mediated inflammatory cascade. GLC might be a therapeutic candidate for the treatment of steatohepatitis developed by alcohol abuse and metabolic disorders.

## Introduction

Chronic liver disease (CLD) is one of the leading causes of death worldwide; around 844 million people suffer from CLD resulting in approximately two million deaths per year (Byass, 2014). At present, obesity and alcohol consumption are the two main causes of CLD (Ntandja Wandji., et al., 2020), promoting the development of alcoholic liver disease (ALD) and non-alcoholic fatty liver disease (NAFLD), respectively. While one of the two factors is always predominant, there is considerable evidence indicating that hazardous alcohol use and obesity-associated metabolic disorders act synergistically to aggravate the progression of fatty liver disease (Aberg*.,* et al., 2020).

The spectrum of liver injury in ALD and NAFLD is quite similar, ranging from steatosis, and steatohepatitis to fibrosis, cirrhosis, and hepatocellular carcinoma (HCC) (MacSween and Burt, 1986; Matteoni., et al., 1999). However, although obesity is an essential contributor to NAFLD, less than 5% of NAFLD patients will ever develop a complicated liver disease (Tsukamoto*.,* et al., 2009). Similarly, even though a strong dose-response relationship between alcohol intake and liver cirrhosis mortality in the ALD population, only 6.26%–10.7% of heavy drinkers develop cirrhosis during their lifetime (Roerecke., et al., 2019). The fact has led to a hypothesis that excessive intake of alcohol or calories is the first hit to induce hepatic steatosis, the early stage of ALD or NAFLD. The second hit is required in the progress of steatohepatitis and cirrhosis (Tsukamoto, Machida, Dynnyk and Mkrtchyan, 2009). Indeed, numerous studies have pointed out that alcohol and obesity synergistically increase the development of hepatic fibrosis and cirrhosis. The result of a French study showed that being overweight (defined as a BM1 ≥ 25 kg/m^2^ in women and ≥27 in men) for at least 10 years was independently associated with the risk of steatosis, alcoholic hepatitis and cirrhosis (Raynard., et al., 2002).

Although a severe liver injury caused by alcohol abuse combined with obesity, the treatment for such a complicated liver disease remains unsatisfactory. Alcohol abstinence, nutrition intervention and controlling body weight are crucial for patients with ALD and NAFLD. However, only a minority of patients with NAFLD achieve weight loss and some regular drinkers still consume alcohol despite medical advice. Thus, pharmacological interventions may be needed. Corticosteroids are the only validated treatment for ALD in the subgroup of patients with severe alcoholic hepatitis (Louvet., et al., 2018). Although various pharmacological approaches aiming to alleviate NAFLD-NASH are currently being examined at different phases of clinical trials (Negi., et al., 2022); no drug is approved by Food and drug administration (FDA) for the treatment of NAFLD.

*Periplaneta Americana*, the American cockroach, has a long history of application for the treatment of various injuries (Wang., et al., 2011; Zhu*.,* et al., 2018). Over the past few years, considerable studies have identified extracts of *Periplaneta* Americana (PAEs) have tissue repair (Yang., et al., 2015), antitumor (Zhao., et al., 2017), antibacterial (Ma et al., 2018), antifungal (Yun et al., 2017), antifibrotic, antiosteoporotic, cardiomyocyte-protecting, and immunity-enhancing efficacy (Zou et al., 2020). However, PAEs have not been explored in clinical applications as a result of the difficulty in purifying the active constituents and the unclear molecular mechanisms. There are only a few representative clinical PAEs prescriptions such as “Kangfuxin solution”, “Xinmailong injection”, and “Ganlong capsule (GLC)” that have been approved to use in clinical treatment until recent years. Particularly, GLC has been widely used in the treatment of chronic hepatitis B in China. The main ingredients of GLC are sticky sugar amino acid, which was identified as the property of immunological regulation and hepatoprotective (Lv, 2017). Sun analyzed the component of water-soluble polysaccharides from cockroaches. The immunological test in normal mice showed the polysaccharide of cockroaches could significantly increase the phagocytic percentage and index of macrophages (Sun et al., 2022). It has been reported that GLC showed a protective effect on chronic liver injury induced by alcohol (Zhang., et al., 2013). However, it remains unclear whether GLC could inhibit steatohepatitis progress driven by both alcohol and obesity.

Here, in this study, we explored the possible protective mechanisms of GLC on liver injury and the progression of steatohepatitis in a mouse model with alcoholics plus a high-fat high-cholesterol diet (EHFD).

## Material and methods

### Chemicals and reagents

Ganlong capsule was purchased from Kunming SINOWAY Natural Pharmaceuticals Co., Ltd (21062101). Palmitic acid was purchased from Sorabio Science and Technology Co., Ltd (Beijing, China).

### Animals and treatments

Male C57BL/6J mice (5–8 weeks old, SPF grade), weighing 20 ± 2 g, were purchased from Zhejiang Vital River Laboratory Animal Technology Co., Ltd (Zhejiang, China) and Guangzhou Ruige Biological Technology Co., Ltd (Guangzhou, China) respectively. The animal welfare practices and animal experimental protocols were strictly consistent with the Guide for the Care and Use of Laboratory Animals, all experimental procedure was officially approved by The Animal Ethics Committee of Jinan University. Animals were maintained in a controlled environment with a temperature of 20°C–26°C and relative humidity of 40–70% under a 12-h light/dark cycle, with standard food and water *ad libitum*.

**FIGURE 1:**
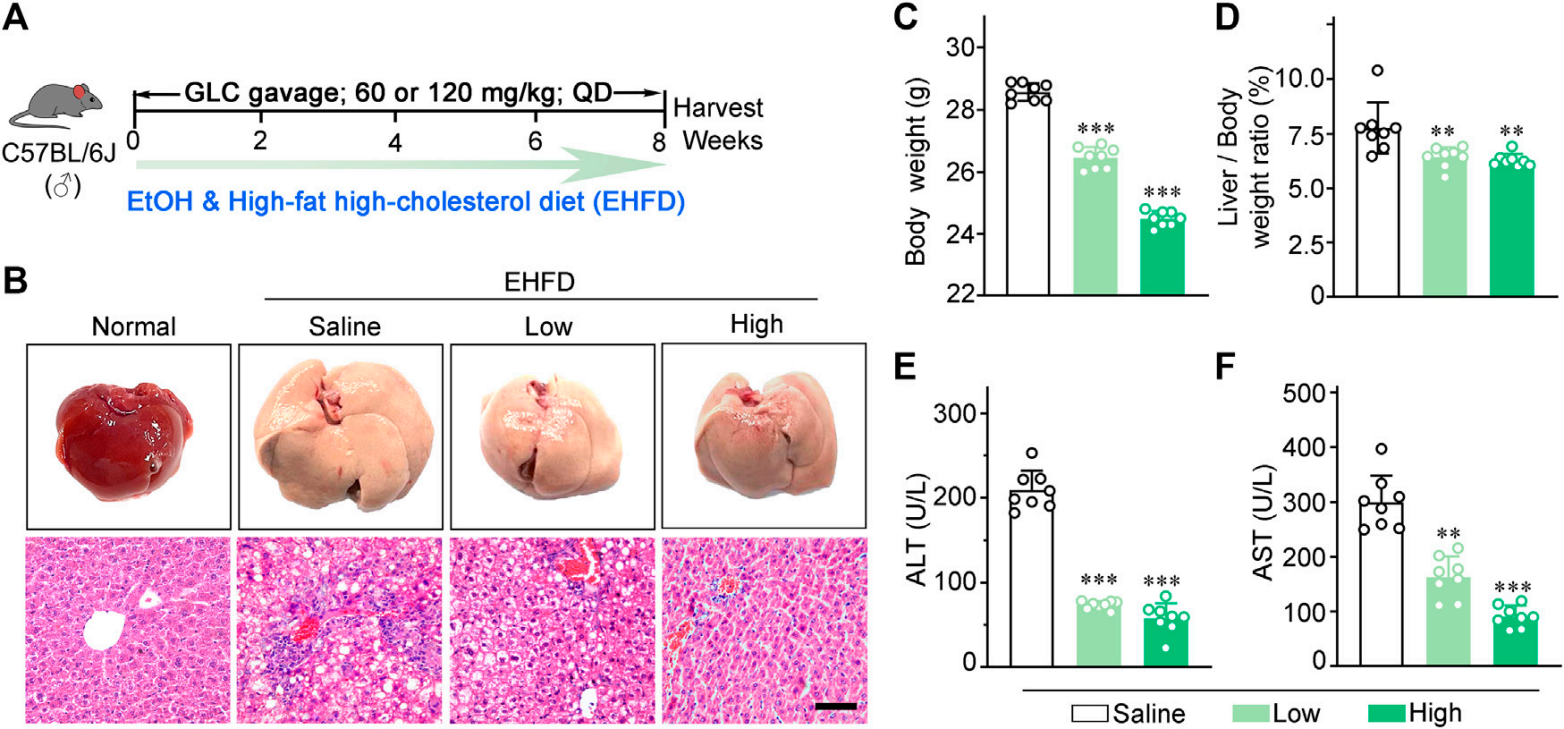
GLC alleviates steatohepatitis induced by EHFD diet. **(A)** Schematic of the liver injury mouse model induced by EHFD diet. **(B)** Representative images of the mouse liver and H&E staining with EHFD treatment. Scale bars, 50 μm. **(C)** The final body weight and **(D)** liver index of EHFD mice (n = 8 for each group). **(E)** Serum ALT and **(F)** AST levels of EHFD mice with or without GLC treatment **P < 0.01 vs. the EHFD mice with the treatment of saline; ***P < 0.001 vs. the EHFD mice with the treatment of saline.

Mice were randomly assigned to the following groups: 1) the control group, which received a standard chow diet (5% fat w/w); 2) the EHFD group, which received a high-fat high-cholesterol diet (containing 17% fat and supplemented with 1.25% cholesterol and 0.5% cholate) which was obtained from Trophic Animal Feed High-tech Co. Ltd (China), with *ad libitum* access to alcohol in drinking water with increasing concentrations of alcohol (1% v/v of alcohol for the first 2 days, 2% from day 3 to day 7, 4% for the second week, and 5% for another 6 weeks); 3) the EHFD + Low group, based on EHFD group, mice were gavaged with 60 mg/kg BW GLC solution per day for 8 weeks; 4) the EHFD + High, based on EHFD group, mice were gavaged with 120 mg/kg BW GLC solution per day for 8 weeks. The control, EHFD mice were given saline at equivalent volumes as the treatment group. The schematic of the animal experiment was shown in Figure 1A. The mice were euthanized at the age of 14 weeks after fasting for 16 h, and the serum and liver tissue were harvested for subsequent measurement.

### Collection of GLC serum

To determine the preventive effect of GLC on liver injury, some normal mice were given GLC treatment with a standard dose according to the clinical use for the patient. GLC-treated group mice were gavaged with GLC (90 mg/kg, twice per day) for 7 days. The control mice were given the same volume of normal 0.9% saline. After 2 h of the last gavage, the mice serum was collected and heat-inactivated at 56°C for 30min, then stored at -20°C for the subsequent experiment (Qin et al., 2021; Zhang, 2021).

### Cell culture and treatments

NCTC1469 and J774A.1 cell line was obtained from the Procell Life Science & Technology Co., Ltd (Wuhan, China) and Newgainbio Co., Ltd (Wuxi, China), and cultured in DMEM supplemented with 10% fetal bovine serum and 1% penicillin streptomycin at 37°C in a 5% CO_2_ humidified atmosphere. We collected portal vein serum from normal male C57BL/6J mice (Ctrl serum) and only GLC gavage-treated male C57BL/6J mice (GLC serum) at a dose of 90 mg/kg BW for 2 h. NCTC1469 cells were treated with GLC serum or other agents when they were 50–60% confluence. GLC serum was added 6h before the treatment of ethanol or palmitic acid.

### Co-culture of hepatocytes and macrophages

NCTC1469 cell lines were seeded in 12-well plates, and different concentrations of GLC serum or Ctrl serum dissolved in serum-free media were pretreated for 6 h when they were 50%–60% confluence. Ethanol (400 mM) was added to the culture medium 6 h later. The supernatant was collected as a condition medium for the macrophages. J774A.1 cell lines were seeded in a 12-well plate at a density of 5 × 10^5^/well for 24 h, then treated with condition medium at 1:2 dilution in serum-free media for 24 h. Cells were collected for subsequent experiments.

### Biochemical analysis

The serum biochemical markers, including alanine aminotransferase (ALT) (Servicebo, #GM1102) aspartate aminotransferase (AST) (Servicebo, #GM1103) triglyceride (TG) (Servicebio, #GM1114) and total cholesterol (TC) (Servicebio, #GM1113) hepatic TG (Sangon Biotech, #D799796) levels were measured with a biochemical autoanalyzer (Rayto, Shenzhen, China). The activity of High-mobility group box1 (HMGB1) in cell supernatant was determined by kits (Elabscience Biotechnology Co., Ltd, Wuhan, China) (Mei mian, #MM-44107M1), and the tissue IL-1β (Proteintech, #KE10003), CCL2 (Proteintech, #KE10006) levels were examined according to the manufactures’ instructions.

### Reactive oxygen species (ROS) analysis

Optimal cutting temperature (OCT)-embedded tissues were fixed in 4% paraformaldehyde. Tissue sections were incubated with either dihydroethidium (DHE, 5 μM, APExBio, #C3807) or 2′,7′-dichlorodihydrofluorescein diacetate (DCFDA, 5 μM, Beyotime, #S0033S) for 30 min at 37°C in a humidified chamber protected from light. Images were acquired by fluorescence microscopy (Germany, Leica).

### Quantitative real-time polymerase chain reaction (qRT-PCR) analysis

Total RNA was isolated by using Trizol reagent (Accurate Biology, Changsha, China) according to the manufacturer’s specifications. cDNA was reverse-transcribed using the RT-PCR system. Real-time PCR was performed by mixing cDNA with primers and SYBR Green qPCR Master Mix (APExBIO, Houston, United States). The sequences of the primers were as follows: *Actb* (forward: 5′-GTG​ACG​TTG​ACA​TCC​GTA​AAG​A-3’; reverse: 5′-GCC​GGA​CTC​ATC​GTA​CTC​C)-3′, *Cd36* (forward: 5′-GAC​TGG​GAC​CAT​TGG​TGA​TGA-3’; reverse: 5′-AAG​GCC​ATC​TCT​ACC​ATG​CC-3′), *Fabp1* (forward: 5′-TGG​TCC​GCA​ATG​AGT​TCA​CCC​T-3’; reverse: 5′-CCA​GCT​TGA​CGA​CTG​CCT​TGA​CTT-3′), *Fasn* (forward: 5′-CTG​CGG​AAA​CTT​CAG​GAA​ATG-3’; reverse: 5′-GGT​TCG​GAA​TGC​TAT​CCA​GG-3′), *Scd1*(forward: 5′-TCT​TCC​TTA​TCA​TTG​CCA​ACA​CCA-3’; reverse: 5′-GCG​TTG​AGC​ACC​AGA​GTG​TAT​CG-3′), *Pparγ* (forward: 5′-ATT​CTG​GCC​CAC​CAA​CTT​CGG-3’; reverse: 5′-TGG​AAG​CCT​GAT​GCT​TTA​TCC​CCA-3′), *Pparα* (forward: 5′-TAT​TCG​GCT​GAA​GCT​GGT​GTA​C-3’; reverse: 5′-CTG​GCA​TTT​GTT​CCG​GTT​CT-3′), *Cpt1α* (forward: 5′-AGG​ACC​CTG​AGG​CAT​CTA​TT-3’; reverse: 5′-ATG​ACC​TCC​TGG​CAT​TCT​CC-3′), *Gss* (forward: 5′-TGT​GCC​CTT​TTA​CCC​TCT​TCC​T-3’; reverse: 5′-TCT​TTG​GAG​TGT​GGG​AAT​GGA-3′), *Gsr* (forward: 5′-AGC​CGC​CTG​AAC​ACC​ATC​TA-3’;

reverse: 5′-GAT​GTG​TGG​AGC​GGT​AAA​CTT​TT-3′), *Gpx3* (forward: 5′-GCT​TGG​TCA​TTC​TGG​GCT​TC-3’; reverse: 5′-CCC​ACC​TGG​TCG​AAC​ATA​CT-3′), *Sod1* (forward: 5′-TTG​GCC​GTA​CAA​TGG​TGG​T-3’; reverse: 5′-CGC​AAT​CCC​AAT​CAC​TCC​AC-3′), *Sod2* (forward: 5′- GGT​GGC​GTT​GAG​ATT​GTT​CA-3’; reverse: 5′- CCC​AGA​CCT​GCC​TTA​CGA​CTA​T-3′), *Cat* (forward: 5′-TCA​CCC​ACG​ATA​TCA​CCA​GA-3’; reverse: 5′-AGC TGA GCC TGA CTC TCC AG-3′), *Cyp2e1* (forward: 5′-ACA​GAG​ACC​ACC​AGC​ACA​AC-3’; reverse: 5′-ATT​CAT​CCT​GTC​TCG​GAC​TGC-3′), *Il-1β* (forward: 5′-CAC​TAC​AGG​CTC​CGA​GAT​GAA​CAA​C-3’; reverse: 5′-TGT​CGT​TGC​TTG​GTT​CTC​CTT​GTA​C-3′), *Il6* (forward: 5′-TAC​CAC​TTC​ACA​AGT​CGG​AGG​C-3’; reverse: 5′-CTG​CAA​GTG​CAT​CAT​CGT​TGT​TC-3′), *Tnfα* (forward: 5′-CTG​AAC​TTC​GGG​GTG​ATC​GG-3’; reverse: 5′-GGC​TTG​TCA​CTC​GAA​TTT​TGA​GA-3′), *Ccl2* (forward: 5′-CCA​CAA​CCA​CCT​CAA​GCA​CT-3’; reverse: 5′-TAA​GGC​ATC​ACA​GTC​CGA​GTC-3′).

### Histopathology assessment

Liver tissues were sectioned and mounted on glass slides then stained with H&E and immunohistochemistry of F4/80 (Abcam, #111101), iNOS (Abcam, #15323), Ly6G (Abcam, #238132), CD86 (CST, #19589). Each sample was observed at a ×400 magnification of microscopic field. The liver cryostat section (Germany, Leica) (5 μm) was stained with Oil red O staining. Images were photographed under a microscope (Germany, Leica). Image Pro Plus 6.0 was used to measure the staining Area.

### Nile red staining

Nile red staining was used to specifically stain the intracellular fat. The cells were fixed and stained with Nile red (100 ng/ml) (APExBio, #B8209) in PBS for 30 min. After being washed thrice with PBS, the cells were examined under a fluorescent microscope (Germany, Leica). Image Pro Plus 6.0 was used to measure the positive staining cells.

### Western blot analysis

Liver tissues were lysed in RIPA lysis buffer (Beyotime) with 1 mM PMSF. An equal amount of protein was separated by SDS-PAGE and transferred to the PVDF membrane. The membranes were washed, blocked and incubated with specific primary anti-rabbit antibodies against CYP2E1 (Proteintech, #19937-1-AP), GAPDH (Cell signaling Technology, #2118S), pNF-kB p65 (Wanleibio, #WL02169), NF-kB p65 (Wanleibio, #WL01273b), TLR4 (PTMBIO, #PTM-5192).

### Dataset analysis

The cDNA microarray data (GEO database: GSE28619) were used for relevant analysis.

### Statistic analysis

The data were presented as the means ± standard deviation (SD). The statistical significance of the difference was analyzed by Student’s t test. *p* < 0.05 was regulated as statistically significant. In this study, the statistical analyses were performed using GraphPad Prism eight software.

## Results

### GLC alleviates steatohepatitis induced by EHFD diet

The picture (Figure 1A) is the schematic of the liver injury mouse model induced by EHFD. H&E staining showed that GLC treatment ameliorated inflammatory necrosis and hepatic steatosis with a dose-dependent tendency (Figure 1B). In addition, GLC exerts a hepatic protective character by decreasing the final body weight (Figure 1C) and liver index (Figure 1D) as well as the serum level of ALT (Figure 1E) and AST (Figure 1F). We also developed a mouse model with alcohol alone to identify the effect of GLC on the chronic liver injury of ALD mice. (Supplementary Figure S1A). The results indicated that the mouse liver injury characterized by hepatocytes vacuolar and ballooning degeneration was alleviated by GLC treatment. (Supplementary Figure S1B). Moreover, the final body weight of ALD mice was decreased while the other was increased with the treatment of GLC (Supplementary Figure S1C). The liver index of ALD was decreased by the administration of GLC (Supplementary Figure S1D). And the serum level of ALT (Supplementary Figure S1E) and AST (Supplementary Figure S1F) was downregulated by GLC administration. Moreover, the lipid accumulation of ALD mice with GLC treatment declined (Supplementary Figures S1G, H). It also showed a declining trend in hepatic and serum TG levels, as well as TC levels in the treatment group (Supplementary Figures S1I, J, K).

### GLC alleviates lipid accumulation by regulating lipogenesis *in vivo* and *in vitro*

**FIGURE 2:**
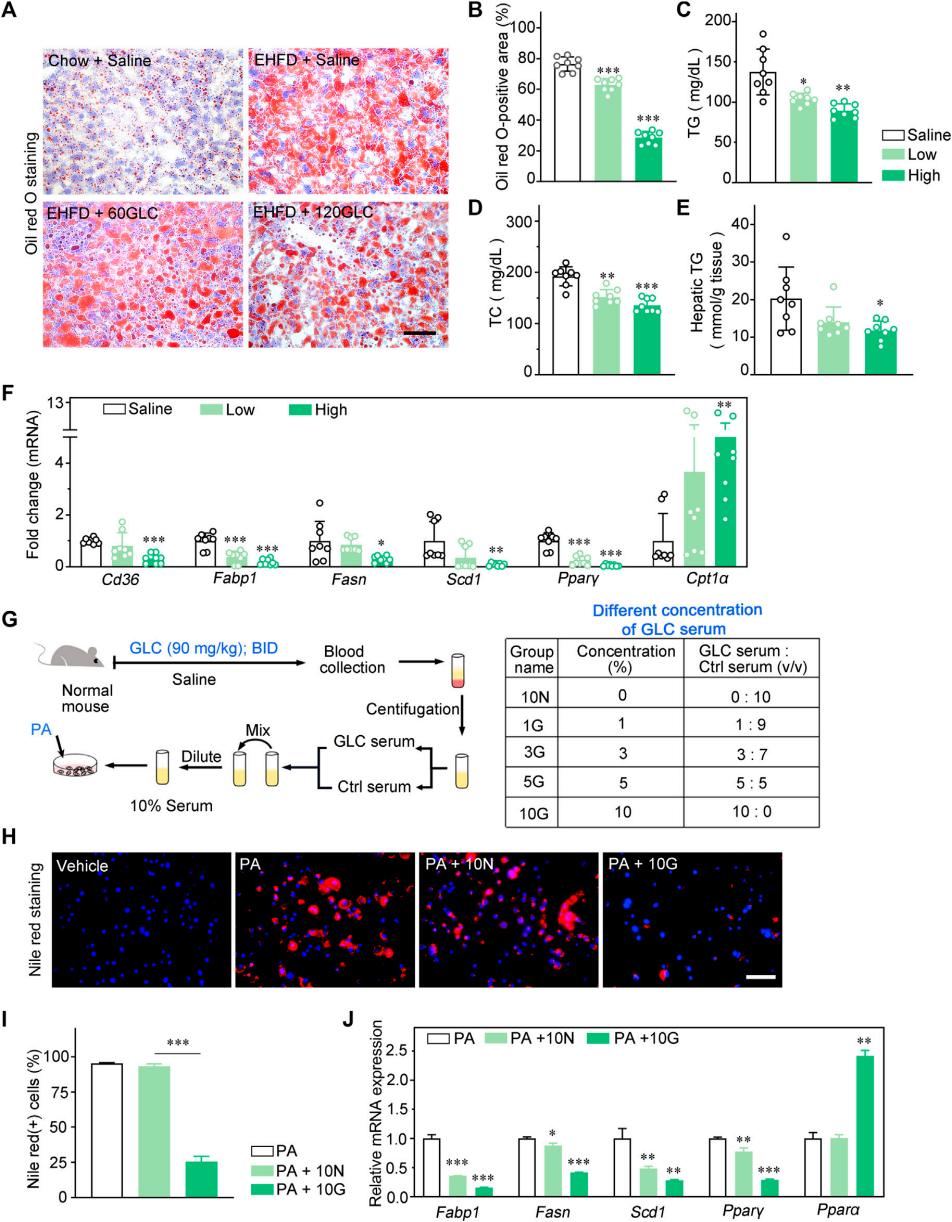
GLC alleviates lipid accummulatlon by regulating lipogenesis *in vivo* and *in vitro*. **(A)** Representative images of Oil red O Staining of the frozen liver sections. Scale bars, 50 μm. **(B)** The statistics of Oil red O-positive areas. **(C)** Serum TG and **(D)** TC levels in EHFD mice. **(E)** Hepatic TG levels in EHFD mice. **(F)** Quanttative PCR analysis of the hepatic mRNA levels of genes related to fatty acid metabolism in mice from the indicated groups. Gene expression was nomalized to actin beta (Actb) mRNA levels (n = 8 for each groups). **(G)** The flow chart of drug-containing serum preparation, the GLC-containing serum group are shown in table. **(H)** Representative images of nile red staining of hepatocytes Induced by EtOH and palmitic acid (PA) with the treatment of GLC-containing serum. Scale bars, 50 μm. **(I)** The statistics of nile red(+) cells. **(J)** Quantitative PCR analysis of the mRNA levels of genes related to fatty acid metabolsm in hepatocytes induced by PA with different concentrations of GLC-containing serum. *P < 0.05; **P < 0.01; ***P < 0.001.

To further investigate the impact of GLC on hepatic steatosis, we analyzed the lipid accumulation of the model mice and therapy groups. The Oil red O staining demonstrated that GLC treatment improved a remarkable accumulation of lipid droplets induced by the EHFD diet. (Figures 2A, B). Consistently, hepatic and serum TG, as well as TC levels, were significantly decreased in GLC-treated mice (Figures 2C–E). Moreover, GLC treatment strikingly decreased the mRNA levels of fatty acid uptake-related and synthesis-related genes (*Cd36, Fabp1, Fasn, Scd1, and Pparγ*) (Figure 2F). Additionally, the mRNA levels of β-oxidation genes (*Cpt1α*) were notably increased in GLC-treated mice compared to EHFD mice (Figure 2F). Considering the complexity of the component in traditional Chinese medicine, GLC-containing serum (GLC serum) and control serum (Ctrl serum) from normal mice was prepared for further study. The main steps were illustrated in the flow chart (Figure 2G). The GLC serum and Ctrl serum were collected for the subsequent experiment *in vitro*. Palmitic acid (PA) induced NCTC1469 cells were applied to study if the different concentrations of GLC serum could improve hepatocytes steatosis. Nile red staining demonstrated GLC serum exerted a dose-dependent decreasing trend of lipid droplets in hepatocytes induced by PA (Figures 2H, I, Supplementary Figure S2A). Consist with the results *in vivo*, GLC serum also declined the mRNA expression levels of genes related to lipogenesis while promoting mRNA expression of genes associated with fatty acid β-oxidation in the PA-induced NCTC1469 cells with different concentrations of GLC serum (Figure 2J, Supplementary Figure S2B). Compared with the vehicle group, Ctrl serum (PA + 10N) also downregulated the expression of genes related to lipid accumulation. The results may be attributed to the protective characteristics of the serum. Wang identified that inactive serum is better for cell growth, and different concentrations of serum had various efficacy for cell culturing (Wang et al., 2018). In our study, GLC serum performed a better effect on decreasing lipid accumulation than Ctrl serum.

### GLC attenuates hepatotoxicity *via* inhibiting oxidative stress injury of hepatocytes

**FIGURE 3:**
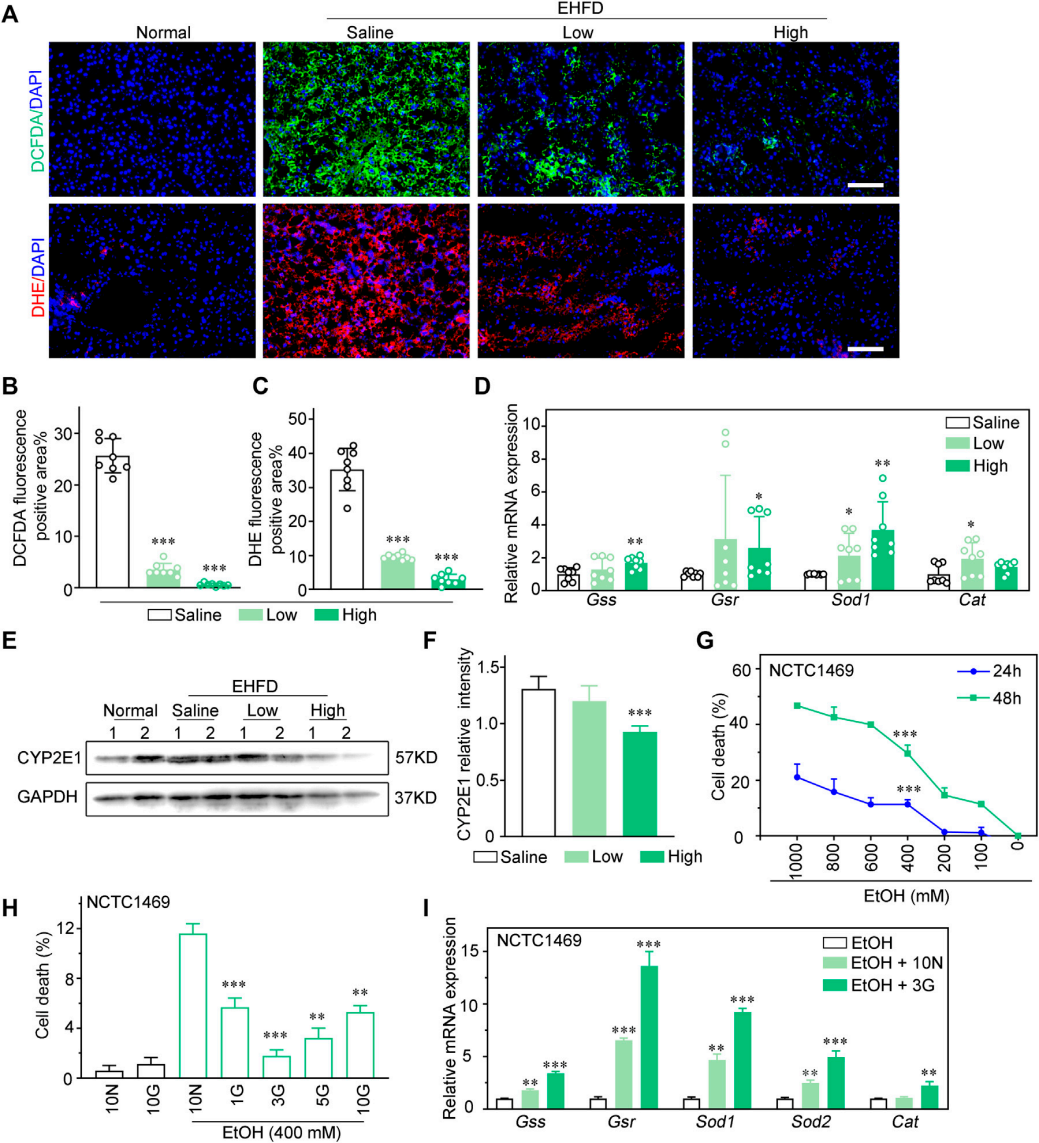
GLC attenuates hepatotoxicity via inhibiting oxidative stress injury of hepatocytes. **(A)** Fluorescence analysis of ROS using DCFDA and DHE dyes in mouse liver sections. Scale bars, 100 μm. **(B)** Statistics of DCFDA and **(C)** DHE positive areas. **(D)** Quantitative PCR analysis of hepatic mRNA levels of genes related to oxidative stress injury. **(E)** Expression of CYP2E1 and GAPDH were examined by western blotting. GAPDH as a loading control (*n* = 2 for each group). **(F)** Relative intensity of CYP2E1. **(G)** NCTC1469 cells were treated with different concentrations of EtOH (0–1000 mM) for 24 h or 48 h. **(H)** NCTC1469 cells were pretreated with different concentrations of GLC serum for 1 h followed by administrationwith EtOH (400 mM). The cell death was analyzed by CCK-8. **P < 0.01 vs. NCTC1469 cells induced by ethanol with treatment of 10% Ctrl serum; ***P < 0.001 vs. NCTC1469 cells induced by ethanol with treatment of 10% Ctrl serum. **(I)** Quantitative PCR analysis of NCTC1469 cells mRNA levels of genes related to oxidative stress injury. The data were obtained from three dependent experiments per group. **P < 0.01 vs. NCTC1469 cells treated with EtOH; ***P < 0.001 vs. NCTC1469 cells treated with EtOH.

Histological analysis revealed that GLC effectively reduces reactive oxygen species (ROS) levels in the mouse model of EHFD (Figures 3A–C). Effects of GLC on mRNA levels of hepatic antioxidant genes (*Gss, Gsr, Gpx3, Sod1,* and *Cat*) were also investigated. The quantitative PCR analysis indicated that the expression of antioxidant genes mentioned above was potently less than those in the GLC treatment group (Figure 3D). CYP2E1 was reported as a major contributor to ROS generation and played a pivotal role in ethanol-induced fatty liver and oxidative stress (Lu and Cedeerbaum, 2008; Lu et al., 2010; Yang et al., 2012). The results identified that the increase of CYP2E1 in the EHFD group was significantly reduced with GLC administration (Figures 3E, F). To further confirm the hepatoprotective property of GLC, we also used GLC serum to pretreat NCTC1469 cells before EtOH exposure. We found that EtOH dose-dependently increased NCTC1469 cell death (Figure 3G), while GLC serum could effectively inhibit EtOH-induced cell death in 3%GLC serum (*P* < 0.0001) (Figure 3H). According to the results, we then performed 3%GLC serum for further study. Quantitative PCR analysis also depicted that ethanol-induced reduction of gene expression related to the antioxidant enzyme was strongly inhibited by GLC serum. These data suggest that GLC serum increased the activity of antioxidant defenses. Furthermore, GLC also exerted a similar effect on injured hepatocytes induced by both EtOH and PA (Supplementary Figure S3).

### GLC suppresses macrophages infiltration and pro-inflammatory cytokines expression *in vivo*

**FIGURE 4:**
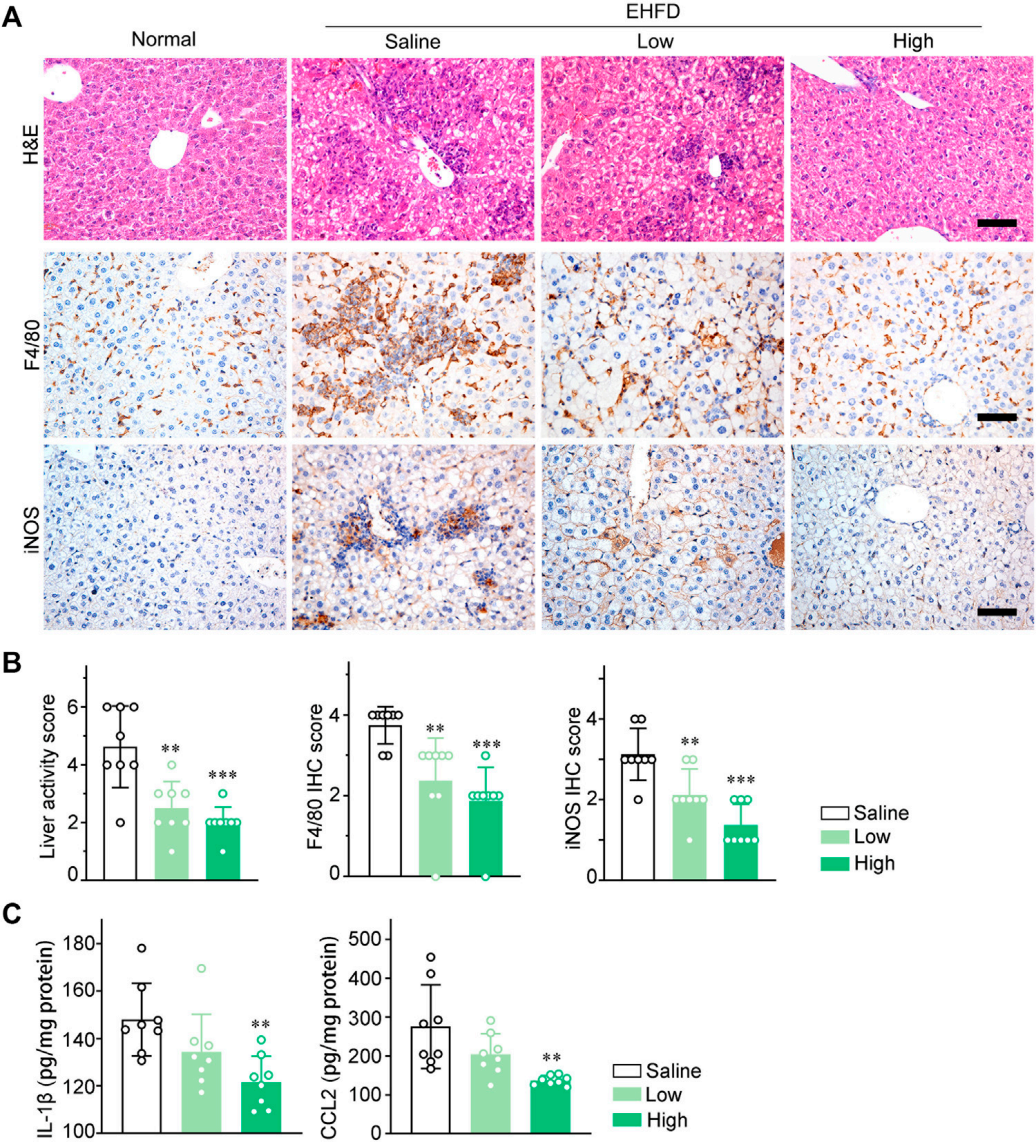
GLC suppresses macrophage infiltration and pro‐inflammatory cytokines expression *in vivo*. **(A)** H&E staining and IHC staining of F4/80, iNOS of mouse liver sections. Scale bars, 50 μm. **(B)** Liver activity score, IHC score of F4/80 and iNOS of mouse liver sections. **(C)** IL-1β and CCL2 levels in liver tissue of EHFD mice with or without treatment of GLC (n = 8 for each group).

To investigate inflammatory response in EHFD model mice, H&E staining of liver sections was performed to compare inflammatory lesions between the model group and the therapy group. As shown in the images (Figure 4A), GLC treatment markedly attenuated inflammatory cell infiltration and hepatocyte necrosis. Ly6G immunohistochemistry (IHC) staining analysis indicated that GLC decreased neutrophil infiltration of EHFD mice (Supplementary Figure S4A). Furthermore, IHC staining of F4/80, iNOS, and CD86 identified that GLC decreased M1 macrophage infiltration (Figure 4A, Supplementary Figure S4A). The results of liver activity score and IHC score of F4/80, iNOS, and CD86 are also consistent with the outcome mentioned above (Figure 4B, Supplementary Figure S4B). Correspondently, we subsequently found that expression of IL-1β and CCL2 were significantly reduced with the treatment of GLC in EHFD model mice (Figure 4C).

### GLC blocks macrophage M1 polarization *via* decreasing HMGB1 released by hepatocytes

**FIGURE 5:**
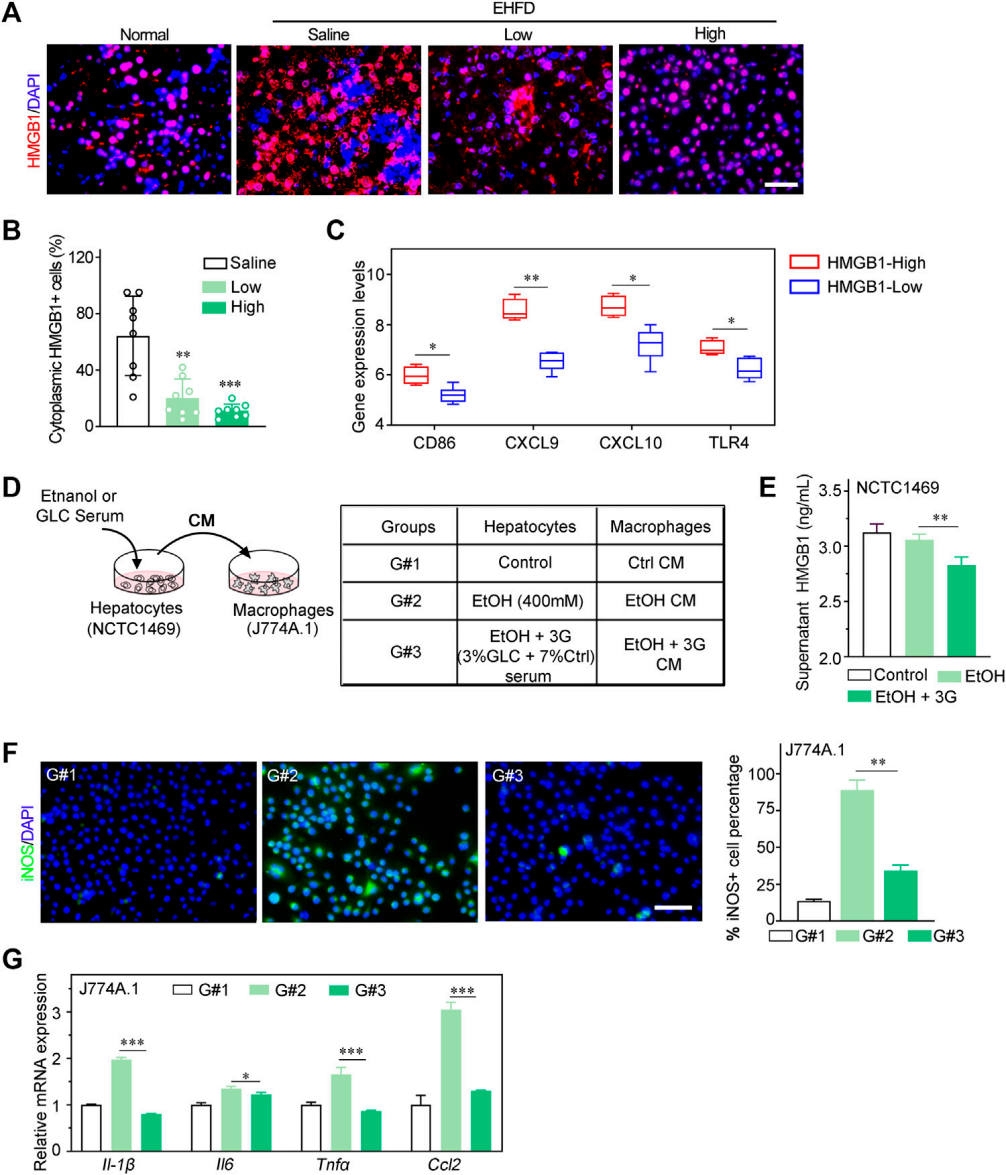
GLC blocks M1 polarization by decreasing HMGB1 released by hepatocytes. **(A)** Immunofluorescence staining of HMGB I in mouse liver tissues. Scale bars, 50 μm. **(B)** Statistics of cytoplasmic HMGB1 positive cells of IF staining sections **(C)** Dataset (GEO database- GSE28619) of patients with alcoholic hepatitis were analyzed. Expression levels of M1 related markers (CD86, CXCL9, CXCL1 0 and TLR4) in HMGB1-High group and HMGB1-Low group. **(D)** Schematic flow chart of co-culture system. NCTC1469 cells induced by EtOH with or without treatment of GLC serum. Supemetant was collected for ELISA and culturing macrophages. The treatment of NCTC1469 cells was displayed in table **(E)** Supernatant HMGB1 levels of NCTC1469 cells induced by EtOH. **P < 0.01 vs. the NCTC1469 cells with EtOH treatment. **(F)** Representative iNOS immunofluorescence staining and statistics of macrophages treated with condition medium (CM) of NCTC1469 cells. Scale bars, 50 μm. **(G)** Quantitative PCR analysis of J774A.1 cell cocultured with NCTCI469 cells. The data were obtained from three dependent experiments per group. *P < 0.05 vs. G#1, ***P < 0.001 vs. G#1.

HMGB1 is recognized as a prototypical DAMP, which was identified to be released by necrotic and apoptotic cells (Scaffidi et al., 2002; Bell et al., 2006). Particularly, HMGB1 is also well known to play a key role in NAFLD, ALD and drug-induced liver injury (DILI) (Khambu et al., 2019). Therefore, we speculated that HMGB1 may act as a mediator of inflammatory response in an early stage of steatohepatitis induced by alcohol consumption and obesity. Consistently, in our study, the translocation of HMGB1 from the nucleus to the cytoplasm and extracellular hepatocytes was significantly lower in the treatment group compared to the EHFD model group (Figures 5A, B). In addition, the ELISA analysis of the supernatant of NCTC1469 cells induced by EtOH indicated that the expression level of HMGB1 was declined with GLC serum treatment (Figure 5E). Apart from that, by using Gene Expression Omnibus (GEO) database (GSE28619), we found that the high expression of HMGB1 was positively related to the M1-related markers (CD86, CXCL9, CXCL10, TLR4) of macrophages (Figure 5C) in patients with alcoholic steatohepatitis (AH), which indicated a closed relationship between HMGB1 expression and M1 polarization of macrophages in the development of steatohepatitis. To further study whether it is HMGB1 released by damaged hepatocytes promotes the M1 polarization of macrophages, a co-culture system was performed to identify the effect of GLC on the interaction between injured hepatocytes and macrophages (Figure 5D). Surprisingly, immunofluorescence (IF) analysis showed a significant reduction of iNOS-positive macrophages when co-cultured with GLC serum-treated hepatocytes induced by ethanol (Figure 5F). Correspondently, the mRNA expression of genes related to M1 markers (*Il-1β, Il6, Tnfα, Ccl2*) was dramatically downregulated in macrophages co-cultured with GLC serum-treated hepatocytes (Figure 5G). Interestingly, we found that GLC not only protected the hepatocytes from oxidative stress and steatosis but also inhibited the M1 polarization of macrophages triggered by LPS. IF staining demonstrated a decrease of iNOS-positive macrophages induced by LPS with the treatment of GLC serum (Supplementary Figure S5A, B). Similar results were detected in PA-treated macrophages. GLC serum reduced iNOS-positive macrophages with PA treatment (Supplementary Figure S5D, E). Particularly, western blot analysis suggested GLC serum alleviates the increase of HMGB1, TLR4, pNF-ΚB (Supplementary Figure S5F, G). Saturated fatty acids could polarize macrophages to an M1-predominant phenotype (Luo et al., 2017), the results of our study identified that PA promoted M1 polarization of macrophages, and GLC serum could suppress the activation of macrophages and block the HMGB1-modulated TLR4/NF-κB pathway. The detection illustrated that GLC exerted a multi-target efficacy in the progression of steatohepatitis. We need a further study to explore the bioactive chemicals of GLC serum and its underlying mechanism of inhibiting the inflammatory response.

### GLC ameliorates the progression of steatohepatitis *via* the HMGB1-mediated TLR4/NF-κB pathway

**FIGURE 6:**
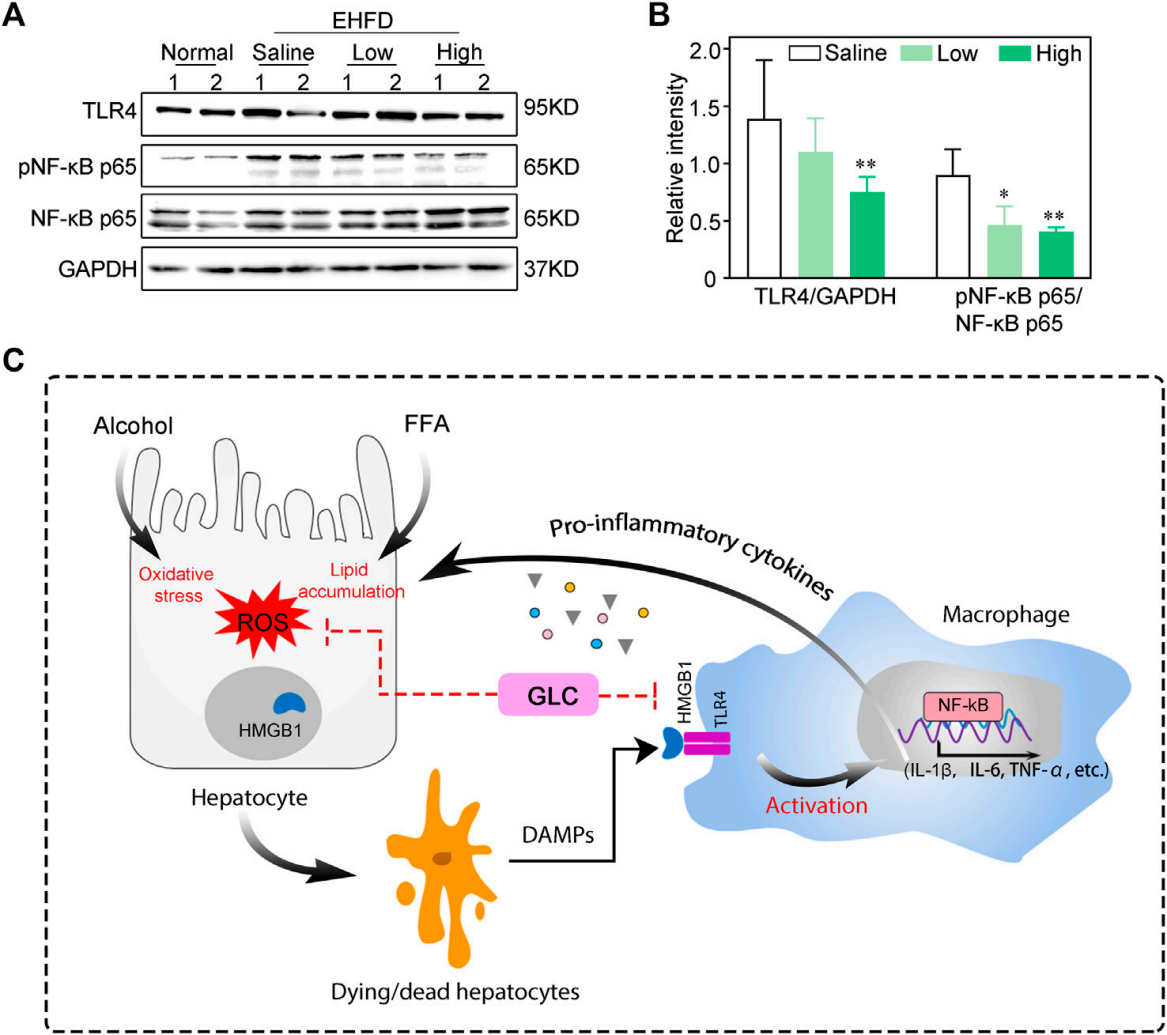
GLC ameliorates progression of steatohepatitis *via* HMGB1-mediated TLR4/NF-KB pathway **(A)** Western blot analysis of indicated proteins expression. **(B)** The relative intensity of indicated proteins expression. The data were obtained from three dependent experiments per group. **(C)** Process of GLC inhibiting HMGB1-mediated macrophage activation by TLR4/NF-KB in the progression of steatohepatitis. Alcoholics and free fatty acids cause oxidative stress injury and lipid accumulation of hepatocytes. The dying/dead hepatocytes release DAMPs - HMGB1 to the extracellular. HMGB1 combined with TLR4 of macrophages to activate the inflammatory response through the NF-KB pathway, which aggravated the hepatocyte injury. GLC exerts hepatoprotective properties by reducing lipid accumulation and oxidative stress. It decreases the HMGB1 in extracellular and blocks TLR4/NF-KB mediated inflammatory cytokines expression of macrophages.

TLR4 is one of the most prevalent and well-studied HMGB1 extracellular receptors (Khambu et al, 2019). The activation of TLR4 initiates a signaling cascade, and stimulates the downstream molecule like NF-kB, inducing the production of pro-inflammatory cytokines (Sung et al., 2013). In our study, western blot analysis demonstrated that GLC reduced the expression of TLR4 and phosphorylated NF-κB in the GLC treatment group compared with the EHFD model group (Figures 6A,B). In the progression of steatohepatitis, alcoholics and HFHC diet cause liver damage by the byproducts of ethanol and lipid metabolism, including ROS. GLC could protect hepatocytes from oxidative stress and steatosis, and reduce extracellular HMGB1 levels, which blocks activation of macrophages *via* TLR4/NF-κB, and the expression of inflammatory cytokines was decreased to alleviate progression of steatohepatitis (Figure 6C).

## Discussion

Excess alcohol consumption and obesity promote the development of steatohepatitis, cirrhosis, and hepatocellular carcinoma (Hart et al., 2010; Naveau et al., 1997; Ruhl and Everhart, 2005; Diehl, 2004). However, the mechanisms underlying the interaction of fat and alcohol in the progression of steatohepatitis are still unclear, and there is lacking effective treatment for such a complex liver disease. *Periplaneta Americana* is a common source of animal medicine with a long history of use in traditional Chinese medicine. Over the past two decades, PAEs have been tested for the promotion of gastric and duodenal ulcer healing (Lu et al., 2019), treatment of hepatic fibrosis (Zeng et al., 2019), inhibition of tumor growth (Zhao et al, 2017), and stimulation of skin wound healing. Many researchers are actively engaged in the analysis and identification of ingredients of PAEs and have made some progress. It has been reported that PAEs contain polysaccharides, peptides, nucleosides, polyols, steroids, terpenes, alkaloids, flavonoids, and isocoumarins. Therefore, PAEs may have multiple targets and biochemical properties. GLC is a kind of crude PAEs, the main biologically active ingredients are sticky sugar amino acid, which was identified as the efficacy of hepatoprotective and immunological regulation (Lv, 2017). Thus, we developed a mouse model of liver injury induced by alcoholics combined with HFHC diet to explore the effect of GLC on steatohepatitis.

GLC is a kind of crude PAEs, PAEs contain polysaccharides, peptides, nucleosides, polyols, steroids, terpenes, alkaloids, flavonoids, and isocoumarins. It has been reported that PAEs could decrease the hepatic TG levels and upregulate the expression of antioxidative enzymes including SOD and GSH in the mouse model of acute liver injury (Zeng et al, 2019). Consistently, our results showed that GLC exerts a protective effect on oxidative stress injury of hepatocytes by increasing the expression of antioxidant genes (*Gss, Gsr, Sod, and Cat*) and reducing lipid accumulation *via* modulating genes related to lipid metabolism (*Cd36, Fabp1, Fasn, Scd1, Pparγ, Pparα, and Cpt1α*). The results suggested that GLC could regulate the oxidative balance and lipid metabolism. In addition, the decrease of HMGB1 of the supernatant of EtOH-induced hepatocytes with the treatment of GLC serum also identified the positive function of GLC in alleviating liver injury. Furthermore, our results also demonstrated that GLC could ameliorate liver inflammation by reducing inflammatory cytokines expression of macrophages *in vivo*.

Mechanically, Our study confirmed that GLC blocked the translocation of HMGB1 in the liver tissue of EHFD model mice, and downregulated the expression of TLR4 and NF-κB. HMGB1 is the prototypical DAMP that could act as telltales of danger by eliciting an early immune response. HMGB1 plays a critical role in initiating and maintaining a chronic inflammatory state in the liver tissue, promoting the progression of steatosis to NASH(Khambu et al, 2019). Similarly, in the rodent model of ALD, alcohol intake elevated the expression of HMGB1, nucleocytoplasmic shutting, and secretion from the hepatocyte. The ROS generated during ethanol metabolism appears to regulate HMGB1 release. Therefore, the treatment of antioxidants in ALD prevented HMGB1 release (Ge et al., 2014). Despite several detrimental factors that can stimulate inflammatory response through diverse pathways mediated by HMGB1, the most important one of the pathways is characterized by the activation of NF-kB. Our results are consistent with the previous study that the inhibition of HMGB1 blocks the TLR4/NF-κB mediated inflammatory response (Tao et al., 2022). Our study indicated that GLC may play an important role in the inhibition of this positive feedback from multiple targets, which is a complicated process that needs further study.

In conclusion, this study suggests that GLC protects the liver against oxidative stress, lipid accumulation, and inflammatory response induced by the EHFD diet. Indeed, GLC suppresses the process of liver injury from hepatic steatosis to steatohepatitis by blocking inflammatory response through the HMGB1-directed pathway. However, we have not figured out the underlying mechanisms of GLC inhibiting the steatosis and oxidative stress of hepatocytes exposed to ethanol and free fatty acid. The precise composition of GLC serum also needs to be identified in the following research. Although the model in this study was established based on the pathogenesis of ALD with obesity, it could not completely mirror the truly human disease.

## Data Availability

The datasets presented in this study can be found in online repositories. The names of the repository/repositories and accession number(s) can be found in the article/Supplementary Material.
