# Supplementary figures and images for: Periplaneta americana extract alleviates steatohepatitis in a mouse model by modulating HMGB1-mediated inflammatory response
Source: Front Pharmacol. 2022 Oct 3;13:995523. doi: 10.3389/fphar.2022.995523 (PMC9583885; doi:10.3389/fphar.2022.995523)

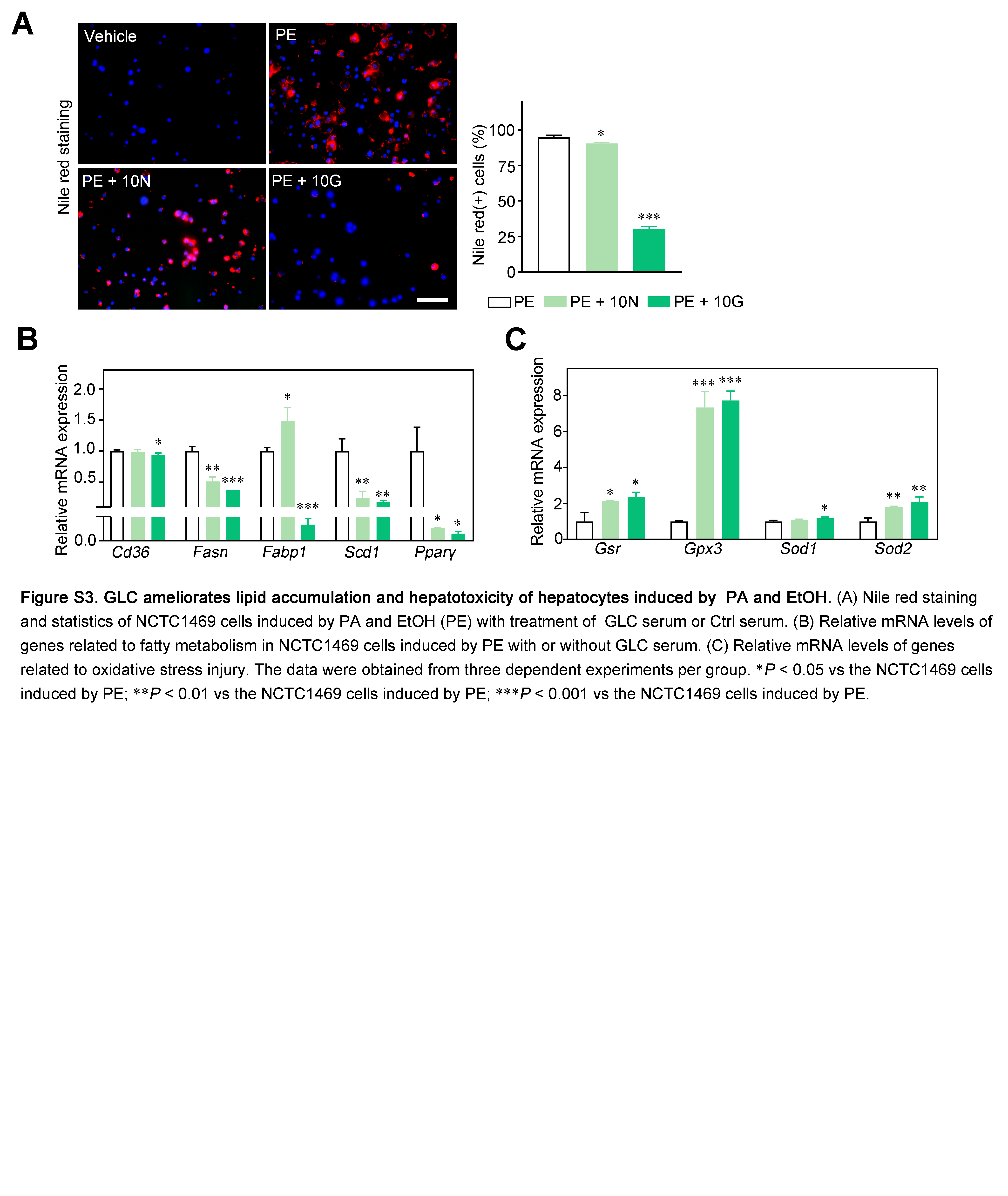

Supplement: Supplementary file 1 [file Image3.tif]

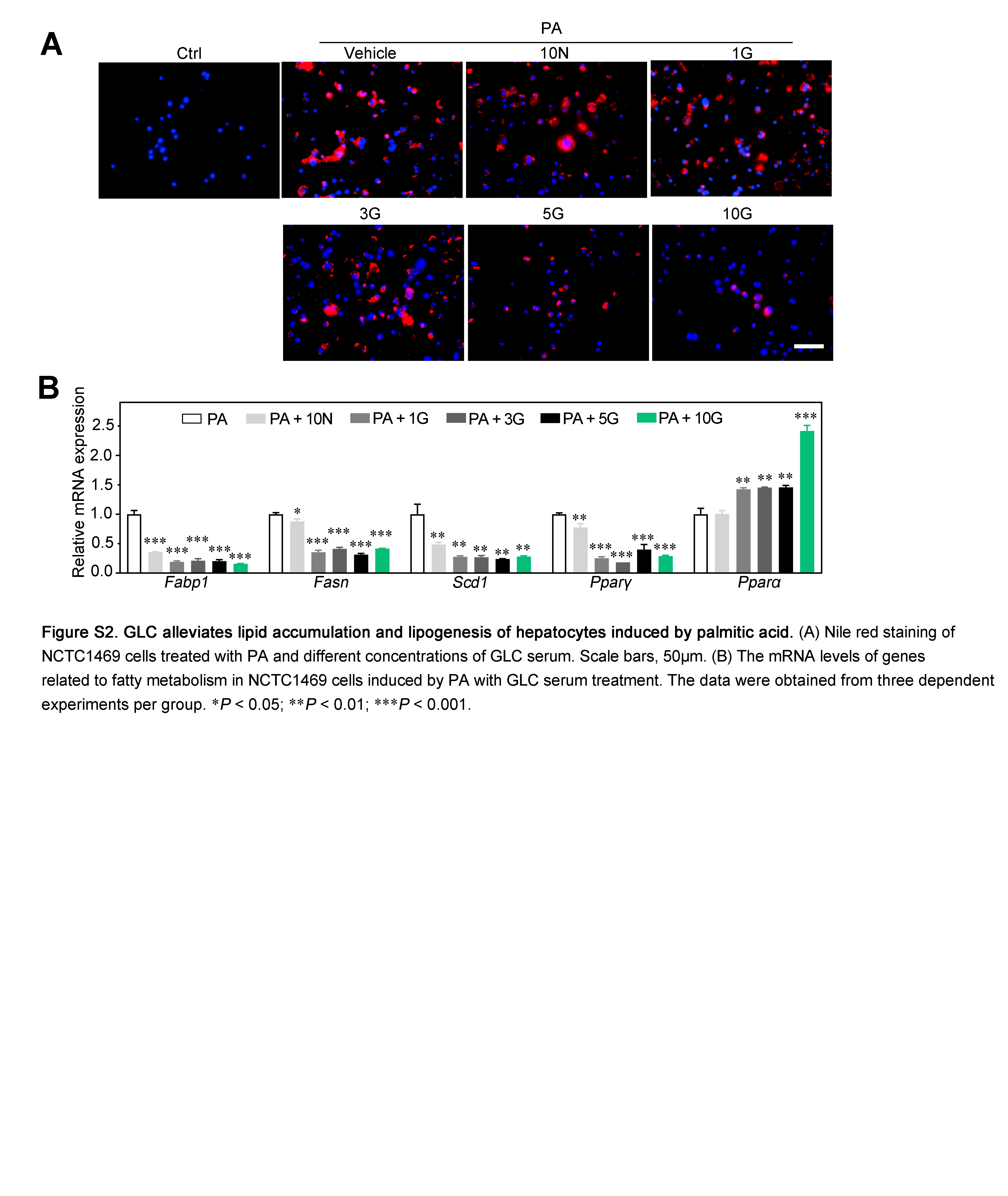

Supplement: Supplementary file 3 [file Image2.tif]

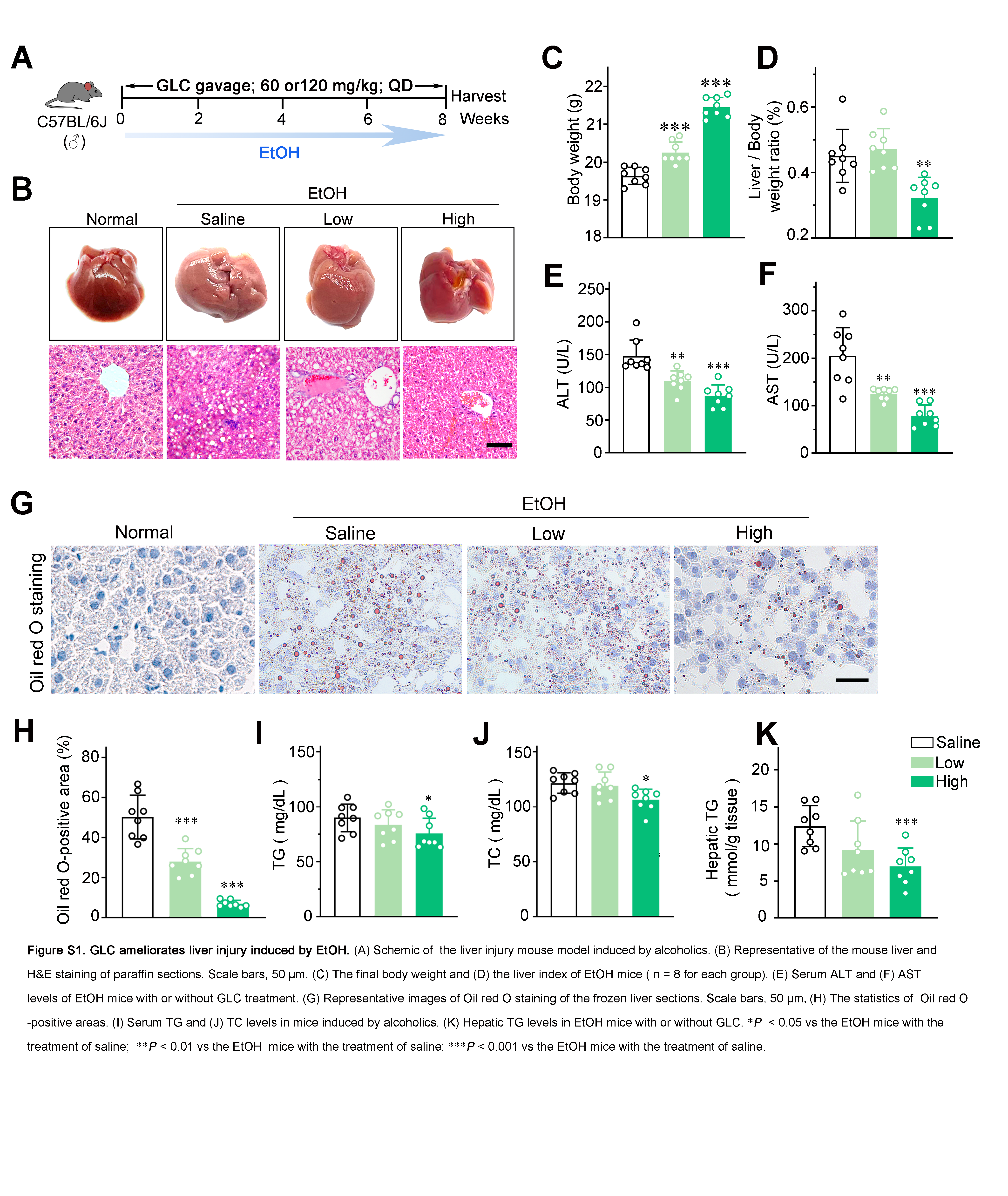

Supplement: Supplementary file 4 [file Image1.tif]

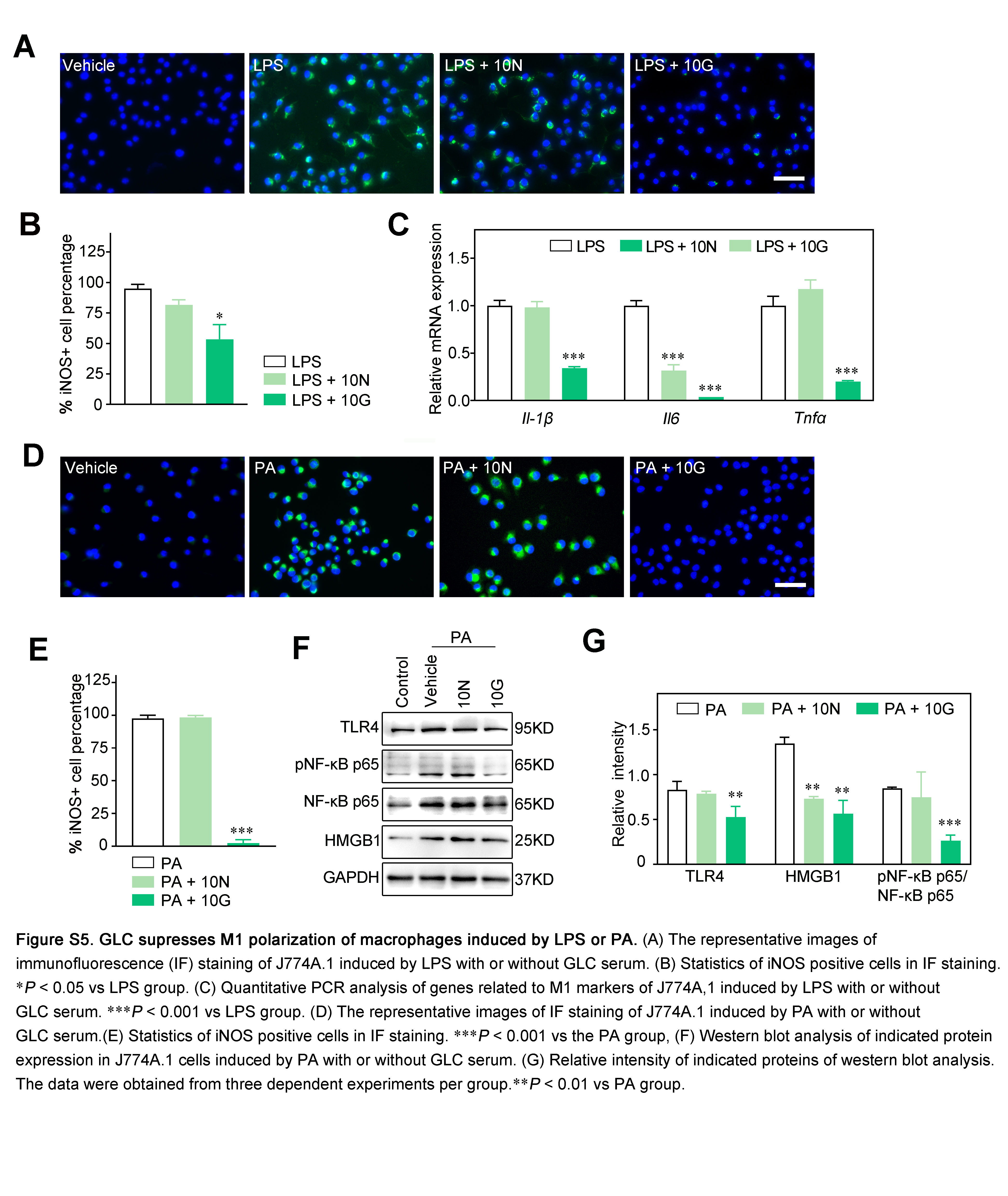

Supplement: Supplementary file 5 [file Image5.tif]
